# A LoRaWAN Multi-Technological Architecture for Construction Site Monitoring

**DOI:** 10.3390/s22228685

**Published:** 2022-11-10

**Authors:** Mattia Ragnoli, Davide Colaiuda, Alfiero Leoni, Giuseppe Ferri, Gianluca Barile, Marianna Rotilio, Eleonora Laurini, Pierluigi De Berardinis, Vincenzo Stornelli

**Affiliations:** 1 Department of Industrial and Information Engineering, University of L’Aquila, 67100 L’Aquila, Italy; 2 Design Methodologies for Embedded Controllers, Wireless Interconnect and System-on-Chip, University of L’Aquila, 67100 L’Aquila, Italy; 3 Department of Civil, Construction-Architectural and Environmental Engineering, 67100 L’Aquila, Italy

**Keywords:** construction site monitoring, wireless sensor network, LoRa, MEMS, RFID, energy harvesting, Internet of Things, remote monitoring, building information modeling

## Abstract

It is necessary to ensure safety in terms of health and accidents through the real-time monitoring of the construction site environment and workers. This problem has become of great importance due to the economic and social implications. Therefore, a sensor-based approach has been found to be beneficial in Building Information Modeling (BIM). Wireless Sensor Network (WSN) technologies are well-suited for the deployment of monitoring systems. A suitable technical solution for node communication in a WSN is Long Range (LoRa) modulation technology. In this study, an autonomous LoRa-based system for the monitoring of a construction site in Lungro, Calabria, Italy, is presented. The spatial monitoring of working personnel is achieved by employing a tracker device with an Inertial Measurement Unit (IMU) and a Global Positioning System (GPS) device. Accesses of personnel and gear to the site are registered using Radio Frequency Identification (RFID) tags equipped with protective gear. Fixed-position solar-powered sensor nodes are also employed for structural monitoring, i.e., movement sensors are used to monitor the variation of scaffolding, building structures, and under-work housing inclinations. Long Range Wide Area Network (LoRaWAN) gateways interface with the nodes and the internet for data exchange, enabling an Internet of Things (IoT) paradigm for the monitoring solution. A comprehensive overview of the workers and structural nodes, along with the RFID access management system and LoRaWAN gateway features, is provided in this article. A description of the web interface is also reported.

## 1. Introduction

Due to the wide variety of building projects and their significance to end customers, the construction sector is recognized as an important part of the foundation of the national economy [1]. However, construction projects are frequently exposed to a variety of unanticipated hazards, making them one of the riskiest industries to work in overall [2]. These scenarios could be fatal if the risk analysis is not appropriately assessed over the life of the construction project [3]. Health and safety monitoring keeps the workplace essentially free from potential injury threats, requiring a supply of appropriate protective plans [4] and gear [5].

Electronic devices can play a key role in assisting with the monitoring of health risk subject applications [6,7,8]. On a construction site, the status of the workers can be difficult to track using conventional methods. To reduce health-related occurrences and other types of mishaps, real-time monitoring can be introduced, both for working personnel and the building structures on the construction site. Moreover, a sensor-based approach can be beneficial in Building Information Modeling (BIM) [9,10], which has recently gained widespread attention in the architectural, engineering, and construction industries, representing the development and use of computer-generated models to simulate the planning, design, construction, and operation of a facility. The personnel is aided by a simulated environment to help identify potential design, construction, or operational problems. The integration of real scenario obtained data can be beneficial in such modeling.

The monitoring of structures has been proposed by using different approaches in recent years. Engineers have collected usable data for structural monitoring using wired and single-hop wireless data-collecting devices [11,12]. The quantity and appropriate location of sensor nodes may be constrained by power and wiring restrictions, which can also raise the cost and complexity of data collecting. The costs associated with deployment and maintenance may also go up. These problems are frequently solved by Wireless Sensor Networks. Adopting the Internet of Things (IoT) principles and using smart nodes to develop adaptable and potent infrastructures for data collection and analysis is the current trend [13,14]. The physical devices containing sensing equipment are connected to the internet, allowing data to be exchanged between many platforms, separating the system implementation technology from a specific stage of the acquisition process to enable increased modularity.

Thanks to numerous new wireless communication technologies, innovative ad hoc hardware device implementations, and significant academic and corporate research efforts in this area, WSNs have seen a remarkable expansion in recent years [15]. They have been used for a variety of unique applications [16]: monitoring the environment [17], industrial control [18], health-related [19,20] and wearable applications [20,21], and early warning systems [22]. The implementation of the ideal SHM and tracking solution for construction sites remains a difficult task for a variety of reasons. Picking the best one from the current options is by no means easy, nor is creating a new system. The technology, topology, reliability, cost, and complexity of the sensing structures are extremely varied. Finding a method that fits them all uniformly is difficult for the aforementioned purpose. In addition, it is difficult to take into account all the contributing factors due to the monitoring topic’s multidisciplinary nature.

Different solutions for construction site monitoring, both wired and wireless, will be briefly reviewed to give a perspective on the state-of-the-art solutions. Structural health monitoring (SHM) is a connected aspect of safety in building areas, so an overview of WSN-based applications will also be reported in the text.

In this paper, a system for construction site safety, both in terms of workers and structural integrity, is presented. The previously presented state-of-the-art solutions are often deployed as standalone systems. In this work, a LoRa-based WSN that includes different safety-enhancing oriented mechanisms is presented in a real scenario implementation. The system can also be efficiently applied in BIM structures. The workers are equipped with a tracker device that allows for seeing the location of the personnel at given intervals of time by means of an online platform. To monitor the accesses of personnel and tools in the construction site, an RFID-based method has been deployed, using tags on hard hats and tools to remotely monitor the element’s position. Accelerometer-based sensor nodes are equipped on the vertical structures of both the scaffolding and buildings to check the variation of inclination in time. These last-mentioned nodes are powered by solar harvesting, while the trackers assigned to every worker are recharged by USB. The access monitoring nodes are also powered by USB. The connectivity between all nodes and the web is provided using the LoRaWAN [23,24] layer, which is accessible using mobile solar-powered gateways which do not require wired internet access. The system is described at the functionality, hardware, and web platform level.

## 2. Related Work

In [25,26], the authors propose a low-cost electronic system for dust particle monitoring in construction sites. Battery-powered nodes with Particulate Matter (PM) 2.5 and PM10 sensors are located in different positions. The devices are based on a microcontroller interfaced with an SDS011 [27] unit and a memory card for local data storage. The system was tested in the city of L’Aquila on a real construction site and reported a highly increased level of particulate during the demolition phases happening in the working days. The work presented in [28] studies a worker safety-oriented wireless system based on sensor nodes placed in an underground construction site to collect hazardous gas levels and environmental conditions, temperature, and humidity. In regions where an abnormal status is detected, an alert is triggered, and the ventilator on site will start automatically removing the hazard. The proposed system can enhance safety in construction and provide reference information in rescue operations by restricting the possible hazard area. In [29], the deployment experience of a WSN in a ground improvement area is reported. The devices implemented are built using off-the-shelf solutions. To monitor ground subsidence, a fuel cell-powered gateway and different sensor devices measuring acceleration, inclination, temperature, and barometric pressure were installed. A poor Global System for Mobile communication (GSM) link, malfunctioning hardware, unknown communication patterns, and closed proprietary software required ad-hoc solutions. To address those problems, the authors state that investigating the deployment of these systems, in the future, will make implementation an easier task. The tracking of workers in construction is a topic that has seen some research efforts in recent years. Video-based approaches have been studied for developing artificial intelligence-based tracking systems. In [30], the authors proposed a scheme for tracking multiple workers on construction sites using video cameras. The challenge of multiple people within the camera’s field of view was addressed by developing a tracking algorithm that required several sample templates of a target and learning a general model applicable to other targets with similar geometry. Another video camera-based tracking system for construction workers was presented in [31], where a Microsoft Kinect device was been employed for image capturing. In [32], the authors presented a sensor-aided intelligent mobile robot system that can help provide high-level navigation functions for indoor construction sites. Using WiFi [33] technology, this system can help increase safety. The goal of the research is the complete integration of the sensor-equipped robots into the WSN to visualize sensed data. A 3D graphical control interface can be used for execution against anomalies that have been detected in buildings. Although this solution can be beneficial in indoor environments, for outdoor applications, where the robots’ paths are less well-defined and the terrain can present roughness, some problems can be encountered. Large area coverage with WiFi can be difficult to achieve in moving applications and the radio link can suffer in presence of obstacles [34]. In [35], a Bluetooth Low Energy (BLE) based system was used to evaluate the use of harnesses at construction sites. The authors deployed BLE beacons for delimiting areas, where the use of a harness was mandatory, in order to detect whether the harness was attached to the corresponding lifeline when the worker entered these areas. The method is based on RSSI measurements. An approach for worker safety based on a wireless bracelet was presented in [36], where the wearable device was used to help the construction worker maintain the safety distance needed for COVID-19 infection preventive rules. In [37], the authors developed a prototype using a hard hat, which is mandatory as a personal security item on construction sites, as a container for electronic devices to monitor the worker’s health state. An Arduino Uno [38] commercial microcontroller board was used as the interface for sensors and radiofrequency (RF) transceivers and a data logging unit was responsible for data storage. In [39], an RSSI-based positioning system was presented. It was based on a ZigBee [40] WSN for construction workers. The research gave an example to demonstrate that a received signal strength intensity (RSSI) based approach could be developed to increase the safety of the construction site. We have, however, no information on an actual implementation. In [41], another ZigBee-based framework of WSN application was proposed that aimed at indoor construction resource tracking. It consisted of a group of stationary and mobile sensor nodes that can communicate with one another, and the locations of the node can be determined by applying the localization method based on the RSSI and trilateration. Locating a mobile device is explained, showing how it is possible to identify the distance of a mobile node with respect to three fixed elements to triangulate the position. Proximity monitoring has been studied with various approaches for construction site monitoring. An RSSI-based system was presented in [42] where a radio signal in the Very High Frequency (VHF) band was used as an alarm when a worker approached the dangerous area of a heavy machine. The researchers proposed another proximity tracking BLE-based system in [43] to help workers to determine possible accidents and avoid them. In [44], the authors used an RSSI-based localization aimed at construction resource tracking by transmitting packets using nodes equipped with an FSK transceiver. The paper reports tests on a small scale area of 8 m by 12 m, with 1 to 2 m accuracy. WiFi nodes and multiple gateways were used in [45] for an RSSI-based tracking system for construction workers. In [46], the researchers showed in a case study how this technology might be applied in construction in an IoT-LoRa [47,48] architecture. The authors deployed LoRa tags in combination with magnetic field sensors on a tower crane to monitor its activity. The results, which can be useful for safety in crane management, show that the technology exceeded the human capacity for the monitoring of data that is available in construction. The sensor tag measured operation hours based on vibration; however, in case the acceleration signal was not strong enough, operation hours were detected using the 3-axis magnetic field sensor using the magnetic field signal of the motor. Another system based on wireless sensor networks for tower crane safety is presented in [49], where the authors used sensing devices mounted on the moving equipment to gather information which was successively analyzed to manage the building operation. Wired sensor nodes communicate with a central station over a short-distance wireless network, and the unit successively forwards data over a cellular network to a remote server to achieve an IoT system. In [50], the authors presented a framework for construction site safety monitoring. The deployed electronic devices use BLE-based location detection that works together with a building information model-based hazard identification method. A cloud-based communication platform was used for user access to data. Potential unsafe areas were defined in a BIM model, and real-time worker locations were acquired to detect possible incidents and to identify locations where workers are exposed to risks. The safety monitoring results were then communicated over the cloud for effective safety management. The devices were of two types, mobile phones for workers and distributed BLE-enabled sensor nodes. The authors achieved a good statistical report for true positive alarm events, showing that this system can be used to provide safety enhancements on a building site. However, using smartphones for tracking workers may not represent the most economical solution as those devices can easily incur damage in such an environment; moreover, some construction companies do not allow smartphones on-site. Therefore this approach should be carefully examined in real scenarios. In [51], the authors proposed a system to locate prefabricated components (PC) on a construction site and monitor their structural status during the installation process. RFID technology and strain sensors were used for data on a construction site, and then those were transmitted to a server using LoRa technology. The cloud-based BIM model of the project was used to store and present the project information and real-time onsite data. RDIF readers obtained information from a tag and used the RSSI to triangulate the position of the element; then, a circular area around the calculated tag position was identified to allow better manipulation of the PC elements. During the installation process, strain sensors obtained the structural data of the steel beams. In SHM applications, sensor data can be used for various applications: forecasting masonry construction deterioration, predicting compressive strength of concrete, helping to forecast the best time for the removal of formwork, vibration and control, crack detection, determination of the construction quality, and identification of various damage typologies. An overview is given in [52], where the authors review wireless IoT technology in the monitoring of civil engineering infrastructure. In [53], a wireless sensor node was presented based on Micro-Electro-Mechanical Systems (MEMS) accelerometers for SHM applications. The objective of the work was to estimate the performances of the low-cost MEMS with a possible application in the permanent monitoring of buildings in seismic-prone regions that are often affected by earthquakes. In [54], a prototype system to control the progress of a building project by tracking moving heavy equipment was presented. The authors used commercial standalone electronic boards to realize a sensor node equipped on a machine. A single mobile node was powered by a Li-Ion coin cell rechargeable battery, requiring the intervention of humans when the charge was too low. A central gateway node collected data from the mobile elements. In [55], a WSN-based system was installed on a construction site with the main aim of monitoring the deformation of three diaphragm wall panels in an underground box during the excavation and building process. Wireless tilt and displacement sensors were installed on the boxes to offer some insights into the real performance of a box corner during the excavation process. The sensor nodes were based on a microcontroller interfaced with an IEEE 802.15.4 2.4 GHz [56] radio unit, which communicates with the gateway. Received data were then logged by a sequent device connected using a Universal Serial Bus (USB). In [57], the authors presented a multi-purpose monitoring system for construction areas. The network included different types of nodes equipped with environmental and structural sensors. Sink nodes were implemented to gather data from the sensor units using a star topology, which was in turn connected to the internet using a cellular network. A description of the operating protocols and transmission reliability was provided. In [58], the authors show a real-case scenario of machine learning-based WSN for movement pattern recognition in construction site fence walls. The nodes were based on ScatterWeb MSB [59] nodes that feature a three-axis accelerometer and an 868 MHz Frequency-Shift Keying (FSK) and Amplitude-Shift Keying (ASK) and communicated data to a datalogger node. The aim was to obtain a classification of a movement event into one of four different possible actions that the fence can be subjected to. The main focus of this paper was the testing and evaluation of an artificial intelligence system for a specific application, for which we found a comprehensive performance evaluation. The authors of [60] presented a combined system including wireless nodes and thermal cameras for the observation of concrete columns in a building. By using thermal imaging, the problem of lighting conditions could be solved, allowing the monitoring of objects in situations where classical photometric methods fail. Wireless ZigBee nodes were used to locate the photography position. Concrete curing management was also dealt with in [61], where the authors applied WSN nodes equipped with thermal sensors to evaluate the state of the process during the construction operations of concrete structure buildings. In [62], the authors presented a plan for the integration of smart sensor nodes based on a WSN employing RFID tags and RF-enabled accelerometric devices in a construction site. A comprehensive analysis of costs for the electronic devices and the complete system was reported, along with the renders from a real BIM model of the site. However, it was not possible for the authors to present real scenario data due to the project being in an early stage of deployment. In [63], the authors proposed a WSN for a rockfall monitoring system based on a LoRa, with solar-powered sensor nodes mounted on rock barriers. The nodes monitored the inclination with respect to the node frame, along with environmental data. The information was then transmitted to the internet to form an IoT system where the user could access all the data through an online platform. A system description was provided, along with a power analysis of the sensor nodes.

## 3. System Architecture

### 3.1. Overview of LoRaWAN

**Figure 1:**
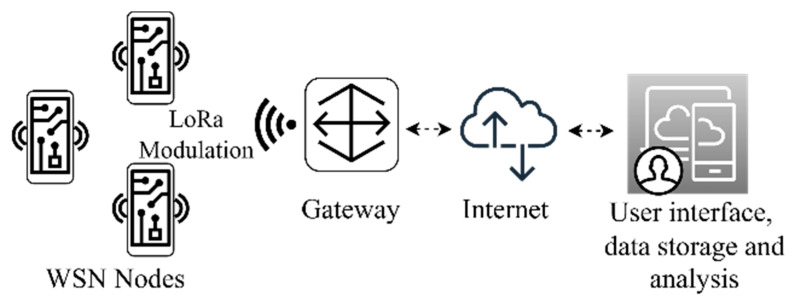
LoRaWAN IoT network general architecture.

Implementing Low Power Wide Area Network (LPWAN) technologies [64] can be advantageous in IoT-oriented WSNs, as is evident from the state-of-the-art studies previously covered. This can help the sensor nodes achieve strong energetic performances, which are essential for energy harvesting in powered devices [65]. An overall affordable deployment is possible using the developing LPWAN technologies [66] which are frequently employed in settings where little data are transferred intermittently, frequently resulting in less complicated transceiver hardware, therefore reducing device prices. It is often the MAC layer deployment that comes with the highest costs for the system [67]. The LoRaWAN MAC layer, regulated by the LoRa Alliance [68], provides free access to the network by only deploying dedicated LoRaWAN gateways. These devices are available commercially in indoor and outdoor certified enclosures. Interfacing LoRaWAN gateways with free web services allow for the creation of a reliable, low-cost network, as will be shown in this paper. In Figure 1, the architecture of a LoRaWAN-based IoT network is shown. The nodes employ a LoRa modulation, a Chirp Spreading Spectrum (CSS) [69]-based Semtech method, to communicate with it over a single hop link.

A comparison of the energetic efficiency of LPWAN IoT devices is presented in [70]. We can see how LoRa modulation stacks up against other IoT technologies in [71], which reports decent coverage even when operating in not optimal link conditions. Using this radio technology, it is possible to develop LPWANs with extensive coverage at low power consumption, which is beneficial for WSNs. For outdoor applications, where connection technologies that we find in the anthropized environment are not available, the aforementioned modulation technology is a good solution [72]. In [73,74], various analyses of security and privacy vulnerabilities in encryption algorithms and physical implementations of LoRaWAN MAC are reported. Many researchers have provided insight into the capability of the LoRa modulation technique for sensor networks and IoT indoor and outdoor applications [75], such as smart cities [76,77], agriculture [78,79], industrial systems [80,81], and biomedical devices [82].

### 3.2. Proposed System

According to the system proposed in this work, the LoRa nodes are of three types: IMU nodes for structures, which also implement a GPS unit for easier localization, trackers for workers, and gate access control RFID-based nodes. Every 60 min, the structure nodes send packets across the LoRa physical layer to the gateway. This component is powered by solar energy harvesting via a solar panel and charge storage battery and is connected to the internet using Long Term Evolution (LTE) and a Subscriber Identity Module (SIM). The tracker node is a Dragino LGT-92 [83] device. It is an open-source GPS tracker based on LoRa technology for data transmission in a compact and low-weight format. It features an IMU with an accelerometer and a gyroscope, all powered by a lithium-ion battery. The LoRa technology used in the device allows it to reach extremely long ranges at low data rates, also working in highly noisy environments due to the spreading spectrum modulation. Because the product is based on an open-source approach, custom development can be implemented on this unit.

**Figure 2:**
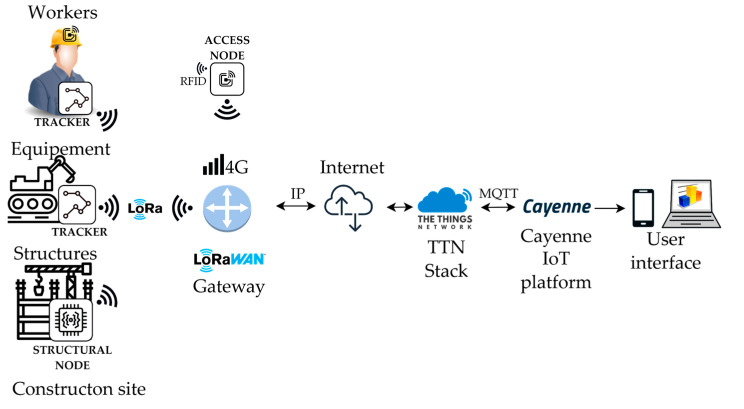
System architecture scheme.

To monitor the accesses within the site, an autonomous system has been developed based on Ultra High-Frequency (UHF) RFID tags. Electronic devices are mounted on the gates of the three sections in which the building site has been partitioned. These nodes use a microcontroller to manage a UHF RFID reader and a radio unit to communicate data to the gateway. Opportune tags are attached to the helmet of every worker. In this way, when passing beneath the gate, the system detects the workers and adds them to the list of the in-site personnel, recognizing the tag through its unique ID. Collected data are sent to a remote server where a real-time view of the access status and records is shown. The same mechanism is applied to construction tools that are operated inside the site. Additionally, on different devices, such as jackhammers, drills, and grinders, the tags are applied to monitor the input and output of instrumentation of the site. The hardware of the nodes will be reported in detail in the following section and the network structure is as follows: after LoRa transmission, The Things Network [84] service manages the packets received at the gateway and employs a payload decoding function to wrap the incoming bytes into a JavaScript Object Notation (JSON) object for data interchange between websites. Each measured quantity is represented by a numerical value and a key-value field in the object. The packets are sent via MQ Telemetry Transport (MQTT) [85] integration to the Cayenne [86] application website, which serves as a user frontend, database. The collected data are displayed on a user dashboard and stored in a database that can be accessed to perform data analysis. The application-specific block scheme of the system architecture is displayed in Figure 2.

**Figure 3:**
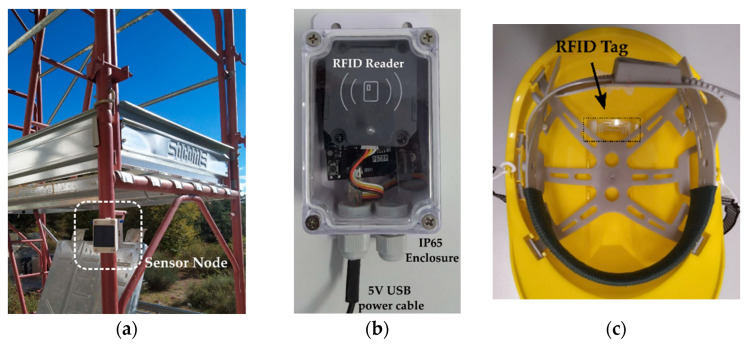
(**a**) A structural node mounted on the side of a scaffolding installation and (**b**) on an entrance gate. (**c**) An RFID tag inside a worker’s helmet.

The WSN’s structural nodes are mounted on the scaffolding poles and on the housing structures which are under maintenance construction operations. In Figure 3, a structural node mounted on scaffolding is shown (Figure 3a), along with a picture of the RFID access reader node (Figure 3b) and worker hard hat device (Figure 3c). The GPS tracker nodes are equipped for every worker at the beginning of the shift and are recharged in charging stations at the end of work hours. The RFID access tags are placed on the unexposed side of the protection helmets in a non-invasive installation.

**Figure 4:**
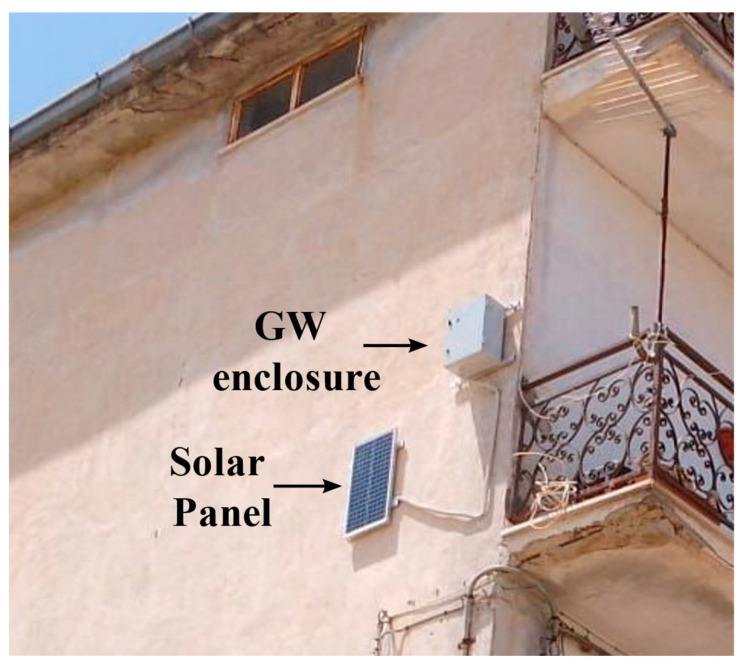
Gateway mounting position.

The LoRa connectivity is provided via two gateways at various locations to allow all sensing devices to have radio communication despite the nodes being located at different heights and positions. The presence of two gateways acts as redundancy in case of the failure of one device. The gateways are positioned so that they can achieve an LTE connection and have enough sunlight to maintain the battery level. Figure 4 shows the mounting position of one of the two gateways (GWs) installed in the WSN.

In order to achieve optimal connection parameters, all of the static devices employ the Adaptive Data Rate (ADR) feature [87,88] of LoRaWAN. Using this mechanism, the network server uses the metadata parameters of the SNR and RSSI of the most recent 20 uplink transmissions. The SNR of the best gateway is used to calculate the margin to determine how much the data rate can be increased or transmission power lowered. This feature allows end devices to modify the transmission parameters in order to achieve minor airtime and obtain a lower power consumption. All the nodes and gateways operate at 868 MHz center frequency in the Industrial Scientific and Medical (ISM) band.

## 4. Developed Structure

### 4.1. Structural Node

**Figure 5:**
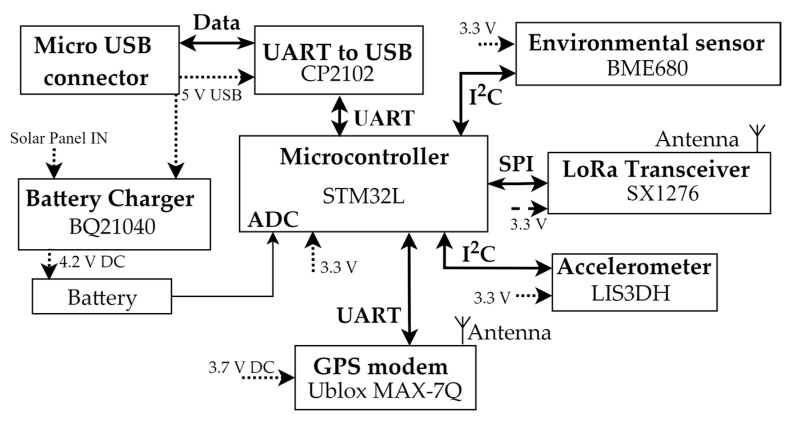
Structural node block scheme.

The structural nodes are electronic systems made up of a coordinator microcontroller, motion and environmental sensors, a UART to USB interface, a GPS modem, a battery management system coupled to a 5 V solar panel, and a power supply and distribution network. The devices come as standalone elements in an enclosure where a custom circuit board and battery are located. The microcontroller is an STM32L [89], especially well suited to battery-powered applications and therefore harvesting-based WSNs, due to its low-power operating mode down to a few microamperes. Multiple Universal Asynchronous Receiver-Transmitter (UART) channels, two Serial Peripheral Interface (SPI) ports, and two Inter-Integrated Circuit (I^2^C) interfaces are all included in the device. For programming and debugging, the USB interface is used, interfaced with the microcontroller using a CP2102 UART to USB circuit [90]. Power is given to this device by the USB bus at 5 V, available at the micro USB connector. A 3000 mAh lithium polymer (LiPo) battery powers the device, and the Analog to Digital Converter (ADC) of the microcontroller is used to read the charge level. A Texas Instruments BQ21040 [91] single-cell charging integrated circuit charges the cell via sun harvesting or straight from the USB connection (TI). The circuits related to the sensor node are powered at 3.3 V by a Low Dropout (LDO) regulator. The movement sensor from STMicroelectronics, MEMS digital output 3-axis accelerometer mode LIS3DH [92], is powered at 3.3 V and connected to the microcontroller via I^2^C. The sensor can operate in different resolution settings, based on the power mode. In normal mode, the resolution is 4 mg/digit at a ±2 g scale. A Ublox MAX-7Q [93] GPS modem is installed on the device to obtain latitude and longitude for positioning status and uses UART to interface with the MCU. The Semtech SX1276 [94] LoRa module is a transceiver for Long Range technology that uses spread spectrum communication, one of Semtech’s proprietary technologies. A Serial Peripheral Interface is used to connect this unit to the microcontroller, powered at 3.3 V. LoRa transceivers do not require extremely complicated internal structures, so these modules offer robust performance at good commercial prices. The high sensitivity of −148 dBm of the module allows communication in low-link strength applications. An ISM flexible antenna, 2JF0115P [95] from 2 J, is used for LoRa communication. Temperature and humidity, along with barometric pressure, are also measured by the structural nodes using a BME680 [96] on the I^2^C bus. The temperature measurement is ±1 °C over the 0–65 °C range. The humidity measurement accuracy is ±3% Relative Humidity (RH) with 0.5% RH typical drift per year, and the barometric pressure accuracy is ±0.12 hPa with ±1 hPa typical drift per year. To ensure that the barometric pressure inside the box stays at the same level as the outside, a compensation valve is used. In Figure 5, a block scheme of the node hardware is shown.

**Figure 6:**
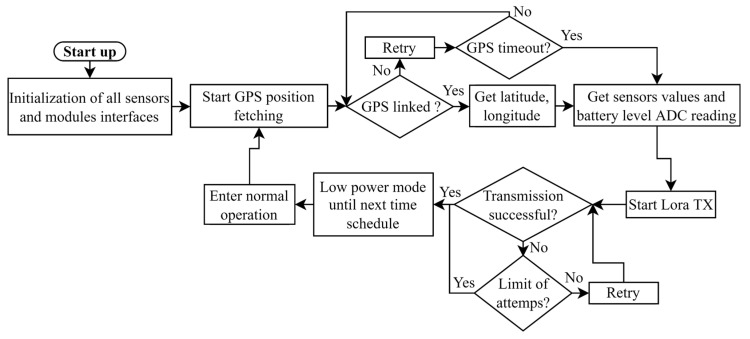
Structural node operation flow diagram.

The structure node functioning is of a sequential nature. When the device is in standby mode, the onboard circuitry enters a low power state to permit minimum power draw and to achieve higher battery duration. Standby is deactivated at predetermined intervals that the installer can choose, and then the data collection process starts. The initial step is to retrieve a GPS position: satellite connections are attempted, and if a GPS response signal cannot be received before a timeout of 30 s, the node continues with sensor reading. Successful links between latitude and longitude are obtained. The LoRa transmission starts following the retrieval of data from the movement sensor and the environmental unit. If the node cannot access the transmission layer after one attempt, it will retry for a total of eight attempts. Metadata containing the RSSI and Signal Noise Ratio (SNR) will also be available in every packet. When data transferring is complete, the node switches back to low-power mode and will go back into standby mode until the next send interval if the LoRa network cannot be accessed during the sending phase after multiple attempts at a gateway connection. In Figure 6, a flow diagram of the structural node is depicted.

The sensor nodes are, in some cases, mounted on structures in high-inclination hillsides. The GPS is used to recover the node in case of displacement. It is, moreover, used to mark the location in the map during the first installation, avoiding manual positioning which becomes complicated for a big number of nodes. In case of scaffolding modification during work operations, a node location should be manually updated, but in this case, it will update itself.

**Figure 7:**
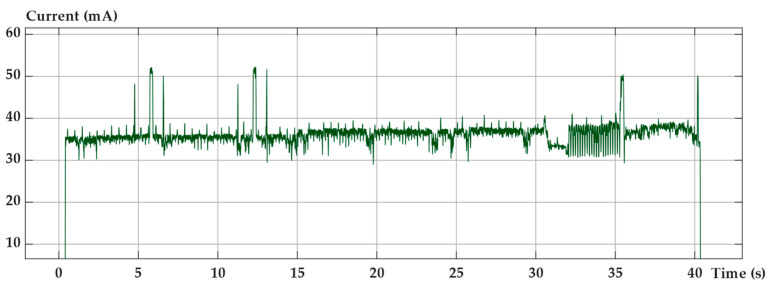
Structural node battery current absorption measured during an activity cycle.

In Figure 7, a graph reporting the current consumption during an active period of data sending attempts is reported. In this graph, the current behavior vs. time is reported for one active period. Peaks due to attempts of GPS signal retrieving are visible in the first section of the trace. The average active current is 35 mA, while in low power mode, between active cycles, the battery supplies an average current of 16 µA.

### 4.2. Worker Tracker

**Figure 8:**
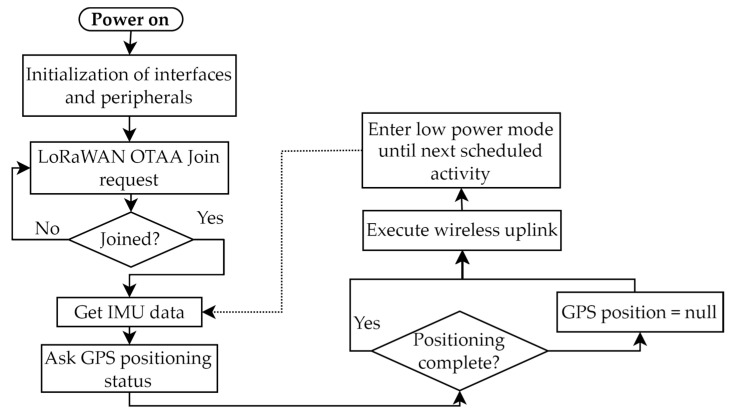
Worker tracking node operation flow diagram in normal mode.

The node Dragino LGT-92 is a microcontroller-based device. It features a native USB 2.0 port. The tracker is powered using a 1000 mAh Li-Ion battery, and the LoRa unit is the SX1276 transceiver. The module works at intervals of 5 min, where the position status is retrieved, along with IMU data and battery voltage. The payload size for location information is 11 bytes long when the IMU data are not requested. If the accelerometer info is requested, the packet is 18 bytes long. The device operates as shown in Figure 8. At first startup, all the onboard peripherals are initialized; this includes the GPS unit, the IMU, and the LoRa transceiver. An Over The Air Activation (OTAA) request is sent through the gateway to LoRaWAN. The device is capable of operating in Wake on Motion interrupt mode, which allows the accelerometer unit to send an interrupt to the microcontroller when the acceleration value along one of the three axes exceeds a threshold. This value can be chosen with a 4 mg resolution up to a maximum of 1020 mg, where g represents the gravitational acceleration. This feature can be used to monitor events such as falls and impacts. Battery lifetime depends on various factors such as GPS signal strength, upload period, and use environment. If the lgt-92 is placed outdoors, with good GPS connectivity, it will take less than 10 s after the first fix. A reference lifetime of a full charge battery on LGT-92-LI, which reports 77 µA current absorption during standby mode, with a transmission period of 5 min is about 19 days.

The tracker includes an emergency mode that the worker can activate by pressing an apposite button for 3 s, not reported in the Figure 8 diagram. An empty packet will be sent, and afterward, a packet with a GPS position will be transmitted. Following, the transmission interval is set at 1 min for 60 packets, and then the device enters normal operation again with the standard interval.

### 4.3. Site Entrance Monitoring

**Figure 9:**
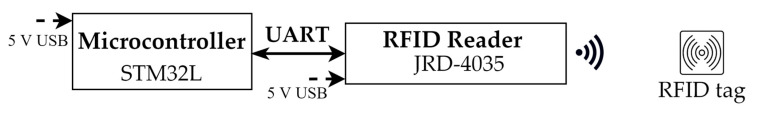
RFID gate access node block scheme.

The nodes use an STM32L microcontroller to manage a UHF RFID reader JRD-4035 [97] that communicates on a UART interface. The reader communicates with the STM32L through serial protocol using a baud rate of 115,200 bits per second and is connected by cable to the microcontroller board, as depicted in Figure 9.

**Figure 10:**
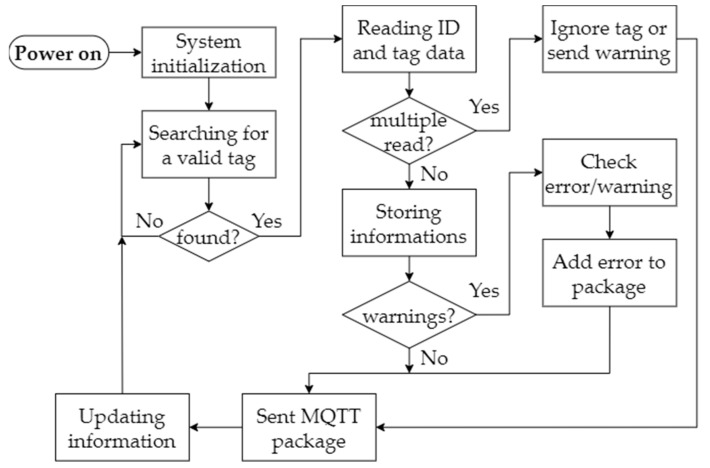
Site entrance monitoring system operating diagram.

Opportune stick tags are attached to every worker’s helmet. In this way, when passing beneath the gate, the system detects the worker and adds them to the list of the in-site workers, recognizing the tag through its unique ID. Collected data are sent to a remote server through LoRaWAN, and using the MQTT protocol integration available on TTN, are then sent to the Cayenne service. When powered up, the reader starts scanning the gate, searching for compatible tags; if it finds a valid tag, the system reads the ID as well as any other information that may be written on it. In this way, it is easy to use this system to differentiate between different work positions and distinguish between workers and tools. The ID is then stored in memory as “in-site” so that a reading again later would mean the exiting of the worker from the site. Of course, to avoid misreading, after a tag is read, there is a certain period in which the reader ignores that tag to avoid errors due to the stationary behavior of the tag carrier. If the same tag is detected multiple times within a short time range (meaning, for instance, a person standing still under the gate), a specific warning is sent to the server notifying it about this issue. Because of safety reasons, there can be a limit on the number of people that can be inside the site at the same time so it can be crucial to detect the access and memorize the workers that enter; if this limit is overcome, the appropriate warning can be sent. A flowchart of the aforementioned system is presented in Figure 10.

### 4.4. LoRaWAN Gateways

**Figure 11:**
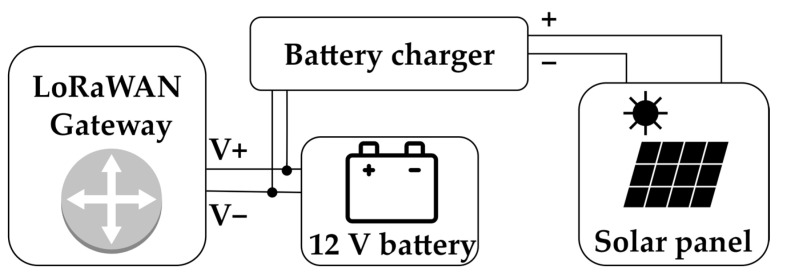
Gateway system block scheme.

The LoRaWAN gateways interface transfers data towards the internet layer through The Things Network service, as previously described. The LTE-enabled gateways are Milesight UG65 [98], powered at 12 V direct current (DC) by a lead-acid battery. An internal 868 MHz antenna is used, with a power level at the output stage for LoRaWAN of 27 dBm and a sensitivity of −140 dBm. The gateways are assembled in a waterproof IP65 enclosure where the supply is also located system. The rated typical total power dissipation of the gateway is 2.9 W, with a specified operating temperature range of the gateways from −40 °C to +70 °C, suitable for external conditions. The solar panel is an NX30P from Energiasolare100 [99] IP65 polycrystalline with a peak power of 30 W, and an open circuit voltage of around 21 V. The battery charge regulator is an EP5 [100], featuring overcurrent and reverse polarity protection, with a 13.8 V operating voltage for battery charge. A PCA12-12 [101] battery from Prime is used for energy storage, powering the gateway through a DC line. In Figure 11, the gateway mounting block scheme is reported.

### 4.5. Network Performances

The adaptive Data Rate feature of LoRaWAN was used for the nodes in the network. The structural nodes report an average SNR of 10 dB and average RSSI of −86 dB, working at a Spreading Factor (SF) of 7 and 125 kHz bandwidth (BW), corresponding to a Data Rate (DR) of 5. The tracker nodes also use the ADR feature with good results, despite being it not specifically suggested for mobile nodes. The average SNR is 10.2 dB with an average RSSI of −87.5 dB. The nodes transmit at an SF of 7 and a BW of 125 kHz. The gate nodes report an average SNR of 10.1 dB and an average RSSI of −85 dBm, working at an SF of 7 and BW of 125 kHz. These performances are possible because the construction site includes two gateways and the area is less than 6200 m^2^, which is well covered by the LoRa network. The Packet Delivery Ratio (PDR) results are better than 90% for all the nodes in the network. The total latency of the information packet is given by the LoRa transmission Time on Air and by the delay introduced in the application server layer. With the aforementioned parameters, the Time on Air relative to the LoRa transmission is less than 100 ms. The data are available on the Cayenne dashboard after an average of 1 s, meaning that the greatest delay is introduced in the application server layer.

### 4.6. Remote Monitoring Platform

A remote monitoring web platform has been implemented using the Cayenne web service to allow the construction site management personnel to observe the operation status of workers and machines, along with structural element parameters. The dashboard is composed of different instances; every instance holds a monitoring panel for each sensor node with related status. To access those data, the user must log in using an enabled email address and password. There are two types of panels: one for structural nodes and one for tracker nodes equipped with workers and machinery. The structural nodes report location as a GPS position, inclination data along three axes as tilt in degrees, battery voltage, the temperature in Celsius, relative humidity, and barometric pressure in Hectopascal. The inclination in degrees is calculated from accelerometric data along the three spatial axes, *acc_x_*, *acc_y_*, *acc_z_*_,_ expressed in $\frac{m}{s^{2}}$ using the following formula, where I_x_ represents inclination along the *x*-axis:

(1) $$I_{x}=\frac{\cos^{-1}\left(\frac{acc_{x}}{\sqrt{acc_{x}{}^{2}+acc_{y}{}^{2}+acc_{z}{}^{2}}}\right)\;\cdot \;180}{\pi }$$

**Figure 12:**
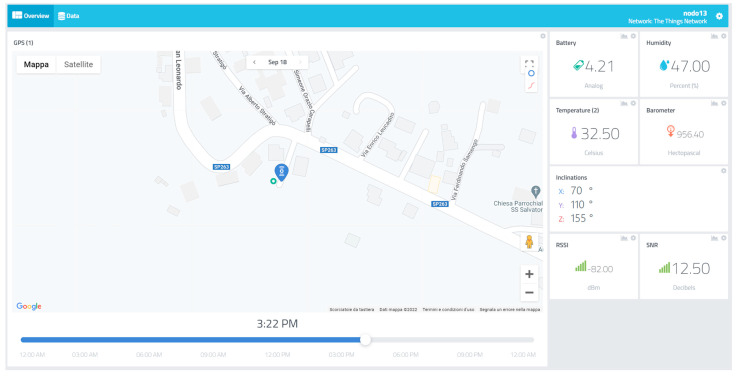
Structural node monitoring dashboard.

**Figure 13:**
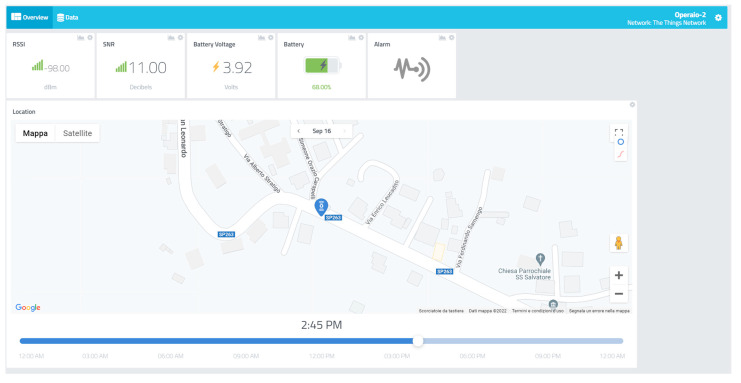
Tracker node monitoring dashboard.

In Figure 12 and Figure 13, some screenshots of the monitoring platform regarding a structural and tracker node are reported, related to a construction site in Lungro, Calabria, Italy. The map, provided using the Google Maps service, reports the last tracked position of the sensor node, identified by the blue marker. For the structural nodes, the environmental parameters, inclinations, and battery voltage are reported on the right side of the dashboard, along with LoRaWAN connection metadata of the RSSI in dBm and SNR in decibels. In the tracker node dashboard, an alarm flag is available to check if a worker or a machine operator has pressed the emergency button on the device and is in need. Using the time slider on the map, it is possible to observe the different locations received at different times of the day to allow a quickly accessible tracking operation. The user can access a historical view of data by accessing the “Data” panel, where a download feature is available to export the history of the node-obtained information. The dashboard can be arranged in a different configuration in case of different user specifications and allows the possibility to insert triggers attached to the received data. These are linked with user-defined alarms. In particular, SMS and e-mails are sent to the site management personnel to activate safety procedures if needed.

**Figure 14:**
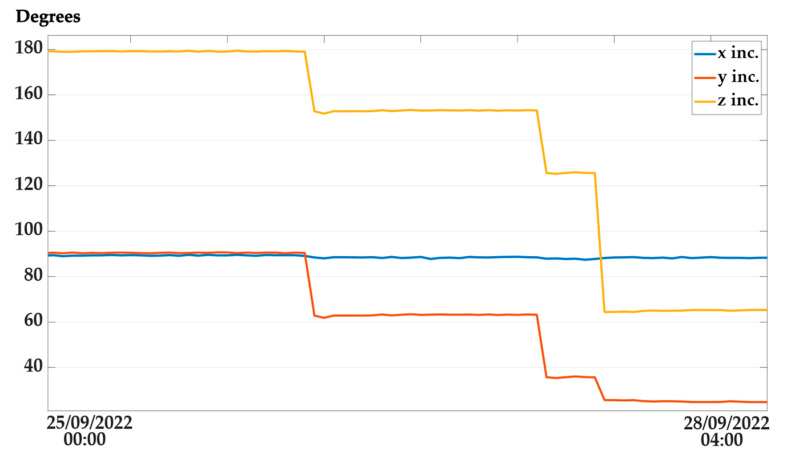
Inclination variation measured from a scaffolding element movement.

In Figure 14, a sample of about three days showing variation in inclinations sensed by the accelerometer of a structural node is reported. The data are relative to an element mounted on a scaffolding pipe, which has varied its position, rotating along an axis of more than 90 degrees.

**Figure 15:**
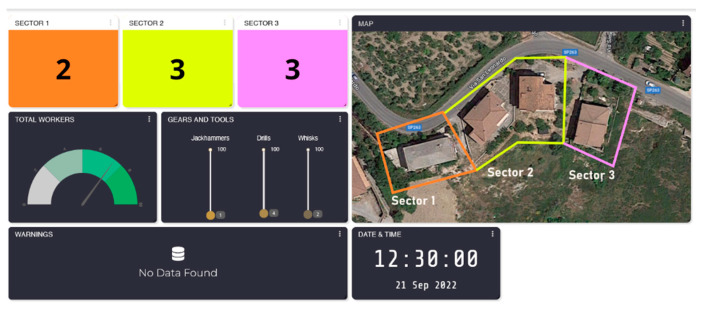
RFID node monitoring dashboard.

Using this system, the construction site managers can benefit from integrating real scenario sensor data into a BIM system to obtain optimized projecting operations, safety enhancement, and general improvement in the site. Inclination data are used by operators to monitor the structural health of scaffolding equipment and critical constructions points. The construction site has been parted into three sections for easier access management and control. Each section is relative to a well-defined zone, and the RFID access monitoring nodes are located at the respective entrance gates. The online monitoring interface allows one to check in real time the location and presence of workers in each section and the count of active tools on the site. In Figure 15, we find a screenshot of the RFID access monitoring dashboard. The sectors are identified by different colors. Exporting historical data regarding accesses is possible for further analysis and integration of data into a BIM model.

The described system has the capability of enhancing building operations by employing smart and autonomous sensor nodes and personnel locating and identification systems, aiming for a more efficient and safety-oriented operating method [102,103]. In Table 1, a brief comparison resume of construction site-oriented monitoring, resource locating, and safety-oriented systems based on the WSN structures that we find in the literature is reported. LPWAN technologies seem to offer a good tradeoff between transmission reliability and energetic performances for the aforementioned application. LoRa modulation technique is one of those particularly suitable for sensor integration in smart building systems. Therefore, the investigation brings useful resources for further progress and deployments.

## 5. Conclusions

The deployment of wired and wireless sensor networks has experienced rapid growth in the last decade for applications in a range of different scenarios. Exploiting sensor nodes’ capacity to deploy BIM mechanisms [104,105] is one of the many applications. Among the different wireless sensor network solutions that were previously reviewed, LoRa technology offers good performance in terms of energy efficiency and coverage. In the literature, we did not find many approaches based on this technology for complete construction site monitoring and BIM-oriented systems. In this paper, a system for the aforementioned application was presented, deploying different sensor nodes and electronic equipment to enable the autonomous tracking of working personnel, tools, heavy machinery, and structural monitoring in a real standalone scenario. In the available research works, mechanisms have been studied as single approaches, while in this work, we deploy mechanisms, which have been proven as potentially helpful for construction site management, in a mixed-type network. The system has been described at the functional and hardware level, with real-case scenario implementation. The personnel of the site can access the data through online dashboards for management operations. Future research and development are related to the current limitations of this work. In addition, the encapsulation of the RFID tags will be modified for better suitability in a rugged environment. Implementation of more robust gate access control must also be carried out, for example, against duplicate data. Moreover, a movement-based alarm will be studied, exploiting the gyroscope feature available in the workers’ trackers.

## Figures and Tables

**Table 1:** Summary of different works that deal with WSN applications in a construction site environment.

Work Reference	Node Type	Type of Data Obtaining Sensors and Peripherals Used	WSN Transmission Technology (Node to Gateway, Node to Node)
[29]	Outdoor, fixed mounting position	Accelerometer, inclinometer, and altitude sensor	GSM
[32]	Indoor, mobile device	Camera, infrared sensor, proximity sensor, ultrasound sensor, and microphone	WiFi
[35]	Indoor	BLE-based devices (RSSI)	BLE
[36]	Indoor and outdoor, wearable device	BLE-based bracelet	BLE
[37]	Indoor and outdoor, mobile device	Pulse sensor, accelerometer, temperature sensor, and GPS unit	FSK and OOK
[39]	Indoor	ZigBee radio unit (RSSI)	ZigBee
[41]	Indoor	ZigBee radio unit (RSSI)	Zigbee
[42]	Outdoor	VHF-based devices (RSSI)	VHF radio
[43]	Outdoor	BLE-based devices (RSSI)	BLE
[44]	Outdoor	916 MHz RF unit (RSSI)	FSK/ASK
[45]	Indoor	RFID tags over WiFi (RSSI)	WiFi and RFID
[46]	Outdoor, fixed mounting position	Magnetometer, accelerometer, and GPS position	LoRa
[49]	Outdoor, fixed mounting position	Load sensor, tilt and angle sensor, displacement sensors, and wind speed sensor	ZigBee and 3G
[50]	Indoor	BLE-based devices used as position sensors	BLE
[51]	Outdoor, mobile devices	Strain sensors, RFID tags	LoRa and RFID
[54]	Outdoor, fixed mounting position	Accelerometer, strain sensor, and GPS position	Bluetooth
[55]	Outdoor, fixed mounting position	Tilt sensor and accelerometer	2.4 GHz IEEE 802.15.4
[57]	Not specified	Accelerometer, displacement sensor, temperature sensor, and smoke sensor	ZigBee and GPRS
[58]	Outdoor	Accelerometer	FSK/ASK
[60]	Outdoor	Infrared cameras	ZigBee
[61]	Indoor	Temperature sensors	ZigBee
[62]	Outdoor	RFID tags and accelerometer	RFID and FSK/ASK
This work	Outdoor and indoor, fixed mounting nodes and mobile devices	Accelerometer, environmental sensor, GPS position, and RFID tags	LoRa and RFID

## Data Availability

The data presented in this study are available on request from the corresponding author. The data are not publicly available due to privacy policies.
